# Adaptive multivariate dispersion control chart with application to bimetal thermostat data

**DOI:** 10.1038/s41598-023-45399-3

**Published:** 2023-10-24

**Authors:** Muhammad Noor-ul-Amin, Muhammad Atif Sarwar, Walid Emam, Yusra Tashkandy, Uzma Yasmeen, Muhammad Nabi

**Affiliations:** 1 COMSATS University Islamabad-Lahore Campus, Lahore, Pakistan; 2 Department of Statistics and Operations Research, King Saud University, Riyadh, Saudi Arabia; 3 Department of Statistics, Brock University, St. Catharines, Canada; 4 Khost Mechanics Institute, Khost, Afghanistan

**Keywords:** Engineering, Mathematics and computing

## Abstract

Adaptive EWMA (AEWMA) control charts have gained remarkable recognition by monitoring productions over a wide range of shifts. The adaptation of computational statistic as per system shift is the main aspect behind the proficiency of these charts. In this paper, a function-based AEWMA multivariate control chart is suggested to monitor the stability of the variance–covariance matrix for normally distributed process control. Our approach involves utilizing an unbiased estimator applying the EWMA statistic to estimate the process shift in real-time and adapt the smoothing or weighting constant using a suggested continuous function. Preferably, the Monte Carlo simulation method is utilized to determine the characteristics of the suggested AEWMA chart in terms of proficient detection of process shifts. The underlying computed results are compared with existing EWMA and existing AEWMA charts and proved to outperform in providing quick detection for different sizes of shifts. To illustrate its real-life application, the authors employed the concept in the bimetal thermostat industry dataset. The proposed research contributes to statistical process control and provides a practical tool for the solution while monitoring covariance matrix changes.

## Introduction

Statistical process control (SPC) has been extensively studied and applied for its simplicity, effectiveness, and capability to detect process deviations^1^. An essential aspect of SPC is identifying and monitoring special cause variations in production processes, which contributes to enhancing process efficiency and product quality^1^. UM), utilize previous observations to enhance sensitivity^2,3^. The variable control charts can be primarily classified into two main categories: memory-less and memory-based. Memory-less control charts rely solely on recent sample information to monitor process parameters, without considering historical statistics. On the other hand, memory-based control charts utilize previous samples to improve its working^1^. Control charting was introduced by Walter A. Shewhart in 1924 which has since become an indispensable instrument in enhancing quality. These charts help identify the appropriate timing for corrective action when a process shift occurs^4^. The commonly used charts include X-bar, R, and S charts, which are effective in monitoring and improving processes. Alternatively, memory-based control charts like the EWMA and CUSUM incorporate past observations to enhance sensitivity^2,3^."

The efficiency of EWMA control charts in detecting small shifts in process parameters has attracted considerable attention in the literature^5–7^. However, traditional control charts assume prior knowledge of the shift magnitude, which is often not the case. To address this limitation, researchers have focused on developing adaptive charting designs that provide improved performance against shifts of various sizes. One such approach is the adaptive EWMA (AEWMA) chart, which combines the strengths of both Shewhart-type and EWMA-type charts seamlessly^8^. By adjusting the weight of previous observations under the error magnitude, the AEWMA chart can detect shifts of different sizes while mitigating the inertia issue. The literature on adaptive control charts continues to advance. For instance, Zhao et al.^7^ utilized adaptive algorithms to analyze dynamic monitoring systems in energy storage systems, specifically voltage difference faults. Arshad et al.^9^ suggested an AEWMA chart that relies on a continuous function to oversee process variance. In industrial settings, there are often scenarios that require the simultaneous monitoring of multiple related quality characteristics. Multivariate statistical process control (SPC) is employed to address these situations. Quality control charts play a crucial role in multivariate SPC^10,11^. Various control charts have been designed to detect variations in the covariance matrix of multivariate normally distributed processes, considering different statistical tests and assumptions about subgroup sizes and data dimensions. However, in practical applications, where subgroup sizes are small and individual observations are considered, additional control charts need to be developed to account for the undefined covariance matrix. Monitoring the variance–covariance matrix in statistical process control is not merely an incremental improvement; it represents a fundamental shift in our ability to ensure process efficiency and product quality. While traditional control charts address univariate variations, the multivariate dispersion control chart enables a comprehensive analysis of multivariate data. This added dimension is pivotal in modern manufacturing and service industries, where processes are inherently complex, interconnected, and influenced by multiple factors. Huang et al.^12^ proposed a control chart based on the trace of the covariance matrix to monitor variations in multivariate normally distributed processes using individual observations. This is the need to crucially design such a control chart that will monitor process variations while considering the multivariate design structure of variables. In recent years, various control charts are suggested monitoring process dispersion shifts both in univariate and multivariate scenarios:^13^ proposed a mixed control chart using both EWMA and CUSUM statistic to construct an EWMA dispersion control chart, Abujiya et al.^14^ has introduced an improvised form of dispersion control chart followed by EWMA statistic only and found effective in identifying small to moderate shifts,^15,16^ has proposed an adaptive version of EWMA chart by using CUSUM accumulate error estimation scheme to estimate the process shift to efficiently monitor process dispersion Zaman et al.^15^ recommended an adaptive control chart using Huber and Tukey function to compute smoothing constant value to determine the proposed EWMA dispersion control chart statistic and found it efficient. Similar efforts are made by the researchers, a few are mentioned as^17–23^, they suggested various modifications while monitoring multivariate cases and designed dispersion control charts.

In response to the constraints observed in current dispersion multivariate control charts, Haq and Khoo^24^ introduced a novel AEWMA control chart known as AEWMA-II. This chart is designed for the surveillance of the covariance matrix in processes that follow a normal distribution. The AEWMA-II chart utilizes an EWMA statistic with an unbiased estimator to estimate the covariance matrix shift and determines the smoothing constant using a proposed continuous function. In this study, a more sophisticated AEWMA multivariate dispersion control chart is suggested to give sensitive detection over a wide range of shifts, named as proposed AEWMA-I. The motivation behind the efficacy of the proposal is the adaptation of smoothing constant value as per shift in the covariance matrix. The suggested control chart plotting statistic uses the smoothing constant as per the estimated shift size and quickly rings the alarm. The proposed AEWMA-I chart overcame the limitations of a high false alarm rate which was due to the higher SDRL than the ARL. The authors addressed this issue by suggesting the new AEWMA-I multivariate dispersion control chart. The suggested design improved the high SDRL issue as well as improved the ARL.

The efficacy is analyzed in terms of smaller run length (RL) profile values like average RL (ARL), standard deviation RL (SDRL), and percentiles at 5th, 10th, 25th, 50th,75th,90th, and 95th in extensive tables through Monte Carlo Simulations. The rest of the paper is structured as: in section "The existing charts" existing control charts are presented, and section "Proposed AEWMA-I control chart" was comprised of the proposed AEWMA I control chart design. Section "Run-length computation" explains the RL computational procedure and performance evaluation is provided in section "Performance comparisons". Real life data set is used in section "Illustrative example" to elaborate on the implementation of the suggested design. At the end of the manuscript, the discussion is wrapped up conclusively in section "Conclusions and further recommendations" with further recommendations along with theoretical contributions and practical implications.

## The existing charts

Suppose we have *p* variable ${\varvec{y}}={\left({y}_{1}, {y}_{2}, {y}_{3}, \dots , {y}_{p}\right)}{\prime}$ with mean vector ${\varvec{\mu}}$ and the covariance matrix ${\varvec{\Sigma}}$, such that,${\varvec{y}}\sim {N}_{p}\left({\varvec{\mu}},{\varvec{\Sigma}}\right).$ Suppose we have the target covariance matrix ${\Sigma }_{0}$ that can vary because of the shifts in the process. This study focused on adapting the value of the smoothing constant with a continuous function. Let the independent, identically distributed (i.i.d.) sequence $\left\{{{\varvec{y}}}_{t}\right\} \forall t>0$, is taken from ${N}_{p}\left({{\varvec{\mu}}}_{0}, {{\varvec{\Sigma}}}_{0}\right)$. Both ${{\varvec{\mu}}}_{0}$ and ${{\varvec{\Sigma}}}_{0}$ are the mean vector and covariance matrix, respectively. Assuming that the process remains in-control state for some unknown time ${t}_{0},$ that is ${\mathbf{y}}_{\mathrm{t}}\sim {N}_{p}\left({{\varvec{\mu}}}_{0}, {{\varvec{\Sigma}}}_{0}\right) \forall t\le {t}_{0}$. After that, the process becomes out-of-control because of an unknown shift $\left({\delta }^{2}\right)$ occurs in ${{\varvec{\Sigma}}}_{0}$, that is ${\mathbf{y}}_{\mathrm{t}}\sim {N}_{p}\left({{\varvec{\mu}}}_{0}, {{\varvec{\Sigma}}}_{1}\right) \forall t>{t}_{0}$, where ${{\varvec{\Sigma}}}_{1}={\delta }^{2}{{\varvec{\Sigma}}}_{0}$ and $\delta >0$. $\delta$=1, $\forall t\le {t}_{0}$ and $\forall t>{t}_{0}$, $\delta \ne$ 1.

Khoo and Quah^25^ proposed a Shewhart control chart to observe the covariance matrix ${{\varvec{\Sigma}}}_{0}$ based on the successive differences between multivariate observations. That is

$${\mathrm{M}}_{\mathrm{t}}=\frac{1}{2}{\left({\mathbf{y}}_{\mathrm{t}}-{\mathbf{y}}_{\mathrm{t}-1}\right)}{\prime} {{{\varvec{\Sigma}}}_{0}}^{-1} \left({\mathbf{y}}_{\mathrm{t}}-{\mathbf{y}}_{\mathrm{t}-1}\right) \forall t>1$$

It can be shown that ${\mathrm{M}}_{\mathrm{t}}\sim {{\chi }_{p}}^{2}, \forall 1<{t\le t}_{0}$, a positively skewed distribution. Experiencing the same thing a control chart with plotting statistic ${\mathrm{M}}_{\mathrm{t}}$ gives biased ARL results on account of its non-normal approach regardless of that ${\mathbf{y}}_{\mathrm{t}}$ has the normal distribution. In the field of SPC, it is a widely adopted practice that numerous researchers have followed, which involves transforming an asymmetrically distributed statistic into a random variable that follows a normal distribution. In what follows, we first transform ${\mathrm{M}}_{\mathrm{t}}$ into a standard normal random variable and then construct an proposed AEWMA-I control chart using this transformed standard normal variable. In the proposed AEWMA-I control chart, a transformation proposed by Quesenberry^26^ is used to normalize the ${\mathrm{M}}_{\mathrm{t}}$, as follows:

$${Z}_{t}={\Phi }^{-1}\left(\mathrm{G}\left({M}_{t};p\right)\right)$$

where $\mathrm{G}\left(.\right)$ is the cumulative distribution function (CDF) of the ${\chi }^{2}$ distribution with *p* degree of freedom and the ${\Phi }^{-1}(.)$ is the inverse CDF of the normal distribution. As ${Z}_{t}\sim N(\mathrm{0,1})$ gives unbiased ARL values for $\forall t\le {t}_{0}$. Let ${E(Z}_{t})\ne 0$ when $\forall t>{t}_{0}$. Thus, it becomes feasible to prepare the conventional mean control chart using $\left\{{Z}_{t}\right\}$ to monitor the erratic fluctuations in the covariance matrix of a multivariate normally distributed process. Let identically dependent distributed $\left\{{Z}_{t}\right\}$, $\forall t>0$ be a sequence of variables based on $\left\{{\mathbf{y}}_{\mathrm{t}}\right\}$. Note that the control charts considered here trigger out-of-control signal only when t > 1 and ${Z}_{1}={\Phi }^{-1}\left({\mathrm{G}}_{{\chi }_{p}^{2}}\left(\frac{{{\mathbf{y}}_{1}}{\prime} {{{\varvec{\Sigma}}}_{0}}^{-1} {\mathbf{y}}_{1}}{2}\right)\right)$.

### The existing EWMA chart

Roberts^3^ proposed EWMA control chart for observing shifts in the mean of a normally distributed process. Haq and Khoo^24^ proposed multivariate EWMA control chart. This chart is helpful to monitor the covariance matrix. Let an EWMA sequence $\left\{{A}_{t}\right\}$ based on $\left\{{Z}_{t}\right\}$, given by

$${A}_{t}=\psi {z}_{t}+\left(1-\psi \right){A}_{t-1}, {A}_{0}=0,$$

where the smoothing parameter $\psi \in (\mathrm{0,1}]$. The EWMA chart reduces to the Shewhart chart when $\psi$ = 1. ${A}_{t}$ is normally distributed with the mean 0 and variance

$${{\varvec{\Sigma}}}_{{A}_{t}}=\frac{\psi }{2-\psi }\left[1-{\left(1-\psi \right)}^{2t}\right] \forall t\le {t}_{0}$$

The term ${\left(1-\psi \right)}^{2t}$ converges to zero, As the time *t* increases. The EWMA chart triggers an out-of-control signal when $\left|{A}_{t}\right|$ exceeds the control limit *L (*> 0*)*, i.e., ${A}_{t}$ <  − *L* or ${A}_{t}$ > *L* to indicate a downward or an upward shift in the covariance matrix of the process. The in-control ARL of the EWMA control chart is controlled by *L*.

### The existing AEWMA-II chart

Haq and Khoo^24^ have suggested an AEWMA-II chart to observe the irregular variations in the covariance matrix of a normally distributed process. The AEWMA-II chart updates the smoothing parameter of plotting statistic according to the estimated size of the shift.

Let ${\widetilde{\delta }}_{t}$ be a biased free estimator of shift $\delta$ at time t. Now

$${\widetilde{\delta }}_{t}=\frac{{\widehat{\delta }}_{t}}{1-{\left(1-\psi \right)}^{t}} ,$$

where

$${\widehat{\delta }}_{t}=\psi {z}_{t}+\left(1-\psi \right){\widehat{\delta }}_{t-1}, {\widehat{\delta }}_{t}=0,$$

and the smoothing constant $\psi$ ranges from 0 to 1 such as $\psi \in (0, 1]$. The plotting statistic of the AEWMA-II chart is

$${K}_{t}= {K}_{t-1}+f\left({\widetilde{\delta }}_{t}\right)\left({z}_{t}-{K}_{t-1}\right),$$

where ${K}_{0}=0$ and $f\left({\widetilde{\delta }}_{t}\right)\in (\mathrm{0,1}]$ such that

$$f\left({\widetilde{\delta }}_{t}\right)=\left\{\begin{array}{c}0.015 \forall {\widetilde{\delta }}_{t}\in (0.00, 0.25]\\ 0.10 \forall {\widetilde{\delta }}_{t}\in (0.25, 0.75]\\ 0.20 \forall {\widetilde{\delta }}_{t}\in (0.75, 1.00]\\ 0.25 \forall {\widetilde{\delta }}_{t}\in (1.00, 1.50]\\ 0.50 \forall {\widetilde{\delta }}_{t}\in (1.50, 2.50]\\ 0.80 \forall {\widetilde{\delta }}_{t}\in (2.50, 3.50]\\ 1.00 \forall {\widetilde{\delta }}_{t}\in (3.50, \infty )\end{array}\right.$$

The AEWMA-II chart triggers an out-of-control signal when $\left|{K}_{t}\right|$ exceeds the control limit *L* (> 0), i.e., ${K}_{t}$ <  − *L* or ${K}_{t}$ > *L* to indicate a downward or an upward shift in the covariance matrix of the process.

## Proposed AEWMA-I control chart

In this section, we examined the suggested AEWMA-I control chart. This control chart is useful for detecting irregular variations in the covariance matrix of a *p*-dimensional multivariate process. The proposed AEWMA-I chart is designed to overcome the limitations of the existing AEWMA-II chart, which exhibits a high false alarm rate due to the SDRL being greater than the ARL. To address this issue, we propose the new AEWMA-I multivariate dispersion control chart, which is based on a continuous function. This mitigates the problem of a high false alarm rate and improves the performance of shift detection. In adaptive control charts, different methods have been suggested for selecting the value of the smoothing constant. Since the size of the shift is generally unknown in advance and varies, it is advisable to consider it as a random variable and estimate it using an appropriate estimator. In our method, we evaluate the magnitude of the shift using an impartial estimator and ascertain the smoothing constant for the proposed AEWMA-I multivariate dispersion control chart through a continuous function. This enhances the design effectiveness in detecting shifts of a diverse magnitude in the covariance matrix.

Let $$\overset{\lower0.5em\hbox{$\smash{\scriptscriptstyle\smile}$}}{\delta }_{t}$$ be the shift estimate at time *t*. Following^27^, we have

$${{\widehat{\delta }}_{t}}^{**}= \frac{{{\widehat{\delta }}_{t}}^{*}}{1-{\left(1-\psi \right)}^{t}} ,$$

where

$${{\widehat{\delta }}^{*}}_{t}= {\psi z}_{t}+\left(1-\psi \right){{\widehat{\delta }}^{*}}_{t-1}$$

where $${{\widehat{\delta }}_{0}}^{*}=0$$ and $$\psi \in (\mathrm{0,1}]$$. The $${\widehat{\delta }}_{t}=\left|{{\widehat{\delta }}_{t}}^{**}\right|$$ to find an estimate of $$\delta$$. Thus, the plotting statistic of the offered control chart is

$${S}_{t}= {S}_{t-1}+g({\widehat{\delta }}_{t})\left({z}_{t}-{S}_{t-1}\right),$$

where ${S}_{t}=0$ and $g\left({\widehat{\delta }}_{t}\right)\in (\mathrm{0,1}]$ such that

$$g({\widehat{\delta }}_{t})=\left\{\begin{array}{c}\begin{array}{c}\frac{1}{24\left[1+{\left({\widehat{\delta }}_{t}\right)}^{-2}\right]} \forall {\widehat{\delta }}_{t}\in (0.0, 1.0]\\ \frac{1}{19\left[1+{\left({\widehat{\delta }}_{t}\right)}^{-1}\right]} \forall {\widehat{\delta }}_{t}\in (1.0, 2.7]\end{array}\\ 1 \forall {\widehat{\delta }}_{t}\in (2.7, \infty )\end{array}\right.$$

Drawing inspiration from the logistic function, where the response function lies within the range of 0–1, we employed a systematic trial-and-error approach. This involved experimenting with various functions, such as logarithmic and exponential functions, along with different constants. We aimed to find an appropriate smoothing constant, denoted as $g\left({\widehat{\delta }}_{t}\right)$, that would render the classical EWMA scheme effective in detecting shifts in the covariance matrix within predefined ${\widehat{\delta }}_{t}$ ranges. The continuous function $g\left({\widehat{\delta }}_{t}\right)$ is used for determining the value of the smoothing constant that improves the efficiency of the proposed control chart. The provided text seems to describe the recommended values of constants for a proposed continuous function in the context of an AEWMA-I chart. The purpose of this function is to improve the ARLs and SDRLs of the AEWMA-I control chart, specifically in the early recognition of shifts in the process. The function $g\left({\widehat{\delta }}_{t}\right)$ plays a crucial role in determining the value of the random variable S_t, which is used as the plotting statistic for the proposed AEWMA-I control chart. The authors have conducted experiments and analysis, and based on their findings, they suggest that specific values for the constant in the function $g\left({\widehat{\delta }}_{t}\right)$ (i.e., 24 and 19) are optimal over certain ranges of δ̂_t (${0.0<\widehat{\delta }}_{t}\le 1.0$ and $1.0<{\widehat{\delta }}_{t}\le 2.7$, respectively). These recommended constant values (24 and 19) have resulted in the proposed control chart functioning as a roughly optimized system, achieving smaller and improved ARLs and SDRLs compared to existing control charts.

The AEWMA-I control chart's working methodology is similar to that of the existing AEWMA-II control chart, as recommended by Haq and Khoo^24^. However, the proposed control chart shows a significant improvement in the Run Length (RL) profiles, indicating that it performs better in detecting shifts in the covariance matrix of the process.

**Decision rule.** Whenever $\left|{S}_{t}\right|>$ *L*, the AEWMA-I control chart gives an out-of-control signal.

### The process parameter is unknown

The underlying process parameter covariance matrix might not be understood in advance in real-world situations. Then, using this dataset, we may estimate the covariance matrix, assuming that trustworthy historical data is available from an in-control process. All *n* observation vectors ${{\varvec{y}}}_{1},\boldsymbol{ }{{\varvec{y}}}_{2},\boldsymbol{ }{{\varvec{y}}}_{3},\boldsymbol{ }\dots ,\boldsymbol{ }{{\varvec{y}}}_{n}$ can be transposed to row vectors and listed in the data matrix **Y** of order (*n* x* p*) as follows:

$${\varvec{Y}}={\left({{{\varvec{y}}}_{1}}^{\boldsymbol{^{\prime}}},\boldsymbol{ }{{{\varvec{y}}}_{2}}^{\boldsymbol{^{\prime}}},\boldsymbol{ }{{{\varvec{y}}}_{3}}^{\boldsymbol{^{\prime}}},\boldsymbol{ }\dots ,\boldsymbol{ }{{{\varvec{y}}}_{n}}^{\boldsymbol{^{\prime}}}\right)}^{\boldsymbol{^{\prime}}}.$$

Then, the unbiased estimator of covariance matrix ${\varvec{\Sigma}}$ is, given by

$$\widehat{{\varvec{\Sigma}}}=\frac{1}{n-1}\left[{{\varvec{Y}}}^{\boldsymbol{^{\prime}}}{\varvec{Y}}-{{\varvec{Y}}}^{\boldsymbol{^{\prime}}}\left(\frac{1}{n}{\varvec{J}}\right){\varvec{Y}}\right]$$

$$=\frac{1}{n-1}\boldsymbol{ }{{\varvec{Y}}}^{\boldsymbol{^{\prime}}}\left({\varvec{I}}-\frac{1}{n}{\varvec{J}}\right){\varvec{Y}},$$

where **I** is the identity matrix of order *n* and **J** is (*n* x *n*) matrix of one’s.

## Run-length computation

In this research, we opted the Monte Carlo (MC) simulation approach to asses the efficiency of the AEWMA-I control chart. The MC simulation method is a well-established and widely acknowledged approach for assessing the run-length characteristics of control charts.

To examine the run-length characteristics, including averages, standard deviations, and percentiles, we performed MC simulations with 50,000 iterations. In each iteration, the AEWMA-I control chart was simulated to observe its performance under different scenarios or conditions. By repeating this process 50,000 times, a robust estimate of the control chart's performance characteristics is obtained. During each iteration, we sampled from a multivariate normal distribution to obtain the necessary data for the control chart. By analyzing the results of these simulations, we were able to calculate the average run length (ARL) and the standard deviation of run length (SDRL) for the AEWMA-I chart. The in-control ARL (${ARL}_{0}=$ 370) and $\psi =$ 0.15. The same is performed for the (${ARL}_{0}=$ 500) by taking and $\psi =$ 0.15 and $p=2$ in Table 1. The respective Table 1 is a comparative picture of existing EWMA multivariate dispersion control chart and existing AEWMA-II multivariate dispersion control chart with the proposed AEWMA-I multivariate dispersion control chart. it is found that for all respective increasing and decreasing dispersion shifts the proposed chart gives outstanding effects with improved ARL and controlled SDRL along with the quantiles at 5th, 10th, 25th, 50th,75th,90th, and 95th. One more performance measure is determined in Table 1 as E(ARL), expected ARL to analyze the picture in a broader spectrum.

**Table 1:** Comparative analysis of existing EWMA and AEWMA-II with AEWMA-I ARL_0_ = 500 and *p* = 5.

δ		ARL	SDRL	E (ARL)	P5th	P10th	P25th	P50th	P75th	P90th
0.25	EWMA	5.26	1.27	5.06	4	4	5	6	7	8
	AEWMA-II	2.35	0.86	2.85	2	2	2	2	3	4
	AEWMA-I (proposed)	**4.44**	**1.31**	**4.64**	**2**	**4**	**4**	**5**	**6**	**6**
0.50	EWMA	13.63	7.27	13.03	6	9	12	17	23	28
	AEWMA-II	7.84	6.9	7.04	2	2	6	11	17	22
	AEWMA-I (proposed)	**10.6**	5.13	**9.66**	**5**	**7**	**10**	**13**	**18**	**21**
0.75	EWMA	82.5	74.19	80.25	16	30	60	111	179	231
	AEWMA-II	58.22	63.31	57.52	2	11	39	83	141	185
	AEWMA-I (proposed)	39.9	23.58	40.39	12	22	37	54	72	83
0.80	EWMA	137.96	129.97	138.9	22	46	99	188	307	398
	AEWMA-II	97.07	107.12	96.57	3	18	64	139	237	311
	AEWMA-I (proposed)	55.94	33.58	54.34	16	31	51	75	101	118
0.85	EWMA	237.03	229.11	237.93	32	74	167	325	534	697
	AEWMA-II	168.77	185.2	168.07	3	34	111	241	411	541
	AEWMA-I (proposed)	82.65	51.66	82.05	23	45	75	111	151	179
0.92	EWMA	480.14	473.16	481.24	57	144	337	663	1090	1430
	AEWMA-II	398.23	445.19	399.03	6	76	258	567	975	1286.05
	AEWMA-I (proposed)	178.44	136.5	179.04	41	82	146	239	354	442
1.00	EWMA	500.81	497.3	500.01	58	147	346	690	1149	1499.05
	AEWMA-II	502.14	553.11	500.40	7	102	331	713	1224	1610.05
	AEWMA-I (proposed)	500.41	483.03	500.44	69	165	360	686	1128	1461
1.03	EWMA	379.47	372.48	378.57	46	113	263	525	864	1131
	AEWMA-II	358.99	395.71	359.89	7	74	236	509	870.1	1147
	AEWMA-I (proposed)	345.97	303.52	347.09	61	132	262	470	738	941
1.05	EWMA	302.74	295.79	301.54	38	91	213	420	684	888.05
	AEWMA-II	272.41	298.34	272.31	6	57	178	385	667	878
	AEWMA-I (proposed)	250.18	205.43	251.08	51	106	197	338	520	653
1.08	EWMA	213.01	206.48	212.61	29	66	150	293	482	622
	AEWMA-II	180.11	193.38	180.01	6	40	120	255	435	565.05
	AEWMA-I (proposed)	163.28	124.75	163.07	37	75	135	219	324	405
1.10	EWMA	170.31	162.61	170.39	24	55	120	233	382	496
	AEWMA-II	140.36	150.01	140.76	5	32	94	198	336	441
	AEWMA-I (proposed)	129.79	94.77	129.76	31	62	109	175	254	314
1.15	EWMA	103.68	97.88	103.55	17	35	73	141	230	301
	AEWMA-II	80.98	84.08	80.66	4	20	56	114	192	249
	AEWMA-I (proposed)	**81.57**	55.87	**81.33**	**20**	**41**	**71**	110	156	188
1.30	EWMA	36.15	30.28	36.25	9	15	27	48	75	96
	AEWMA-II	26.92	26.16	26.99	2	8	19	38	62	79
	AEWMA-I (proposed)	**33.62**	22.51	**33.65**	**9**	**16**	**29**	**46**	**64**	76
1.75	EWMA	9.83	6.09	9.86	4	6	8	13	18	22
	AEWMA-II	6.73	5.55	6.79	2	2	5	9	14	18
	AEWMA-I (proposed)	**9.86**	**6.2**	**9.85**	**3**	**5**	**8**	**13**	**18**	**22**
3.50	EWMA	3.16	1.33	3.18	2	2	3	4	5	6
	AEWMA-II	2.37	0.89	2.35	2	2	2	2	3	4
	AEWMA-I (proposed)	**2.9**	**1.37**	**2.99**	2	2	2	**4**	**5**	**6**

Significant values are in bold.

The values of *L* (threshold) of all three charts EWMA, AEWMA-I, and AEWMA-II are given in Table 2. The run-length characteristics of the AEWMA-I chart with different *p* are given in Table 3 when $\delta$ of any magnitude enters the process covariance matrix. Additionally, to depict the overall conduct of the outcomes a short discussion is given by

- When $\psi$ and $\delta$ are fixed, with an increase in the value of *p*, both ARL and SDRL show a tendency to decrease, and vice versa. For instance, from Table 3 with fixed $\psi$ = 0.15, $\delta$= 0.95, and *p* = 2, 3, 4, 5 the respective ARL = (237.34, 188.16, 157.01, 134.43) and SDRL = (213.39, 163.58, 134.59, 114.47) at ARL0 = 370. This shows that the sensitivity of the control chart increases with an increase in the value of the *p.*
- Table 2 presents the values of threshold (*L*) when ARL_0_ = 370, $\psi$ = 0.15, one can observe an increasing pattern in the value of *L* with an increase in the *p.* This shows a wider control limit with the increase in the p.
- When $\delta$ decreases or increases, both the ARL and SDRL values decrease due to the heightened magnitude of $\delta$ in the process dispersion, elucidating the sensitivity of the suggested chart. For instance, from Table 3 shifts like $\delta$ = (0.95, 0.90) with $\psi$ = 0.15 gives the ARL = (237.34, 117.22) and SDRL = (213.39, 86.90), whereas the shifts like $\delta$ = (1.05, 1.10) with $\psi$ = 0.15 gives the ARL = (207.36, 112.37) and SDRL = (177.60, 85.06) for the *p* = 2 and ARL0 = 370. The same pattern is observed at *p* = 3, 4, and 5.

**Table 2:** Values of *L* for all control charts for ARL_0_ = 370, $\psi$ = 0.15.

	*p*			
	2	3	4	5
EWMA	0.9165	0.9215	0.9249	0.9269
AEWMA-II	0.9823	0.9928	0.9978	1.0026
AEWMA-I	0.2148	0.2181	0.2203	0.2217

**Table 3:** The proposed AEWMA-I RL for ARL_0_ = 370 and $\psi$ = 0.15 at diverse dimensions.

$\delta$	2		3		4		5	
	*L* = 0.2148		*L* = 0.2181		*L* = 0.2203		*L* = 0.2217	
	ARL	SDRL	ARL	SDRL	ARL	SDRL	ARL	SDRL
0.05	2.05	0.21	2.00	0.00	2.00	0.00	2.00	0.00
0.10	2.47	0.55	2.01	0.09	2.00	0.00	2.00	0.00
0.15	2.95	0.76	2.15	0.37	2.00	0.05	2.00	0.01
0.20	3.46	0.94	2.45	0.61	2.06	0.25	2.00	0.05
0.30	4.60	1.44	3.26	1.02	2.53	0.73	2.17	0.43
0.40	6.32	2.51	4.38	1.55	3.40	1.21	2.76	0.94
0.50	9.33	4.69	6.16	2.69	4.69	1.92	3.81	1.56
0.60	14.92	8.65	9.55	5.22	7.05	3.66	5.62	2.79
0.70	25.56	15.68	16.65	10.39	12.21	7.60	9.58	5.87
0.80	48.14	30.73	33.40	21.89	25.15	16.91	20.05	13.80
0.85	71.97	48.03	50.82	34.21	39.51	26.96	32.09	22.47
0.90	117.22	86.90	86.13	62.99	68.91	50.07	57.20	41.89
0.92	151.91	121.29	113.31	87.33	90.75	69.43	76.24	57.90
0.95	237.34	213.39	188.16	163.58	157.01	134.59	134.43	114.47
0.97	317.89	302.00	276.88	260.64	246.13	232.69	219.94	206.33
1.00	370.25	352.14	370.37	358.69	370.01	360.90	370.21	363.32
1.03	273.53	246.38	252.80	222.24	237.23	202.39	222.06	187.51
1.05	207.36	177.60	179.51	146.03	161.49	126.83	147.86	112.34
1.08	140.57	111.18	116.82	86.65	101.42	72.60	90.42	62.92
1.10	112.37	85.06	92.22	66.20	78.41	54.05	69.65	46.95
1.15	72.92	52.63	57.17	39.31	48.12	32.08	42.16	27.39
1.20	51.12	35.74	39.53	26.75	33.04	21.75	28.45	18.28
1.30	30.44	21.22	22.94	15.24	18.86	12.15	16.14	10.03
1.40	20.73	14.36	15.42	10.01	12.61	7.79	10.90	6.41
1.50	15.33	10.40	11.48	7.24	9.43	5.55	8.13	4.50
1.75	9.04	5.90	6.87	3.96	5.73	3.03	5.03	2.48
2.00	6.46	3.98	4.96	2.69	4.20	2.07	3.73	1.70
2.50	4.24	2.36	3.36	1.60	2.91	1.21	2.64	0.97
3.00	3.30	1.67	2.70	1.09	2.42	0.78	2.25	0.59
3.50	2.81	1.25	2.39	0.78	2.20	0.52	2.10	0.36
4.00	2.55	1.00	2.22	0.57	2.10	0.36	2.04	0.23
5.00	2.27	0.65	2.08	0.33	2.03	0.18	2.01	0.10
6.00	2.14	0.47	2.04	0.21	2.01	0.10	2.00	0.05
7.00	2.09	0.35	2.02	0.14	2.00	0.06	2.00	0.03

## Performance comparisons

In the field of SPC, the performance of a control chart is commonly assessed by analyzing its run-length profiles, ARL, SDRL, and percentiles. In this study, we follow the same approach and utilize run-length profiles as a benchmark for comparison. To evaluate the effectiveness of the suggested AEWMA-I control chart, we compare it with the existing EWMA and AEWMA-II control charts proposed by Haq and Khoo^24^. The existing AEWMA-II chart was designed to monitor the covariance matrix of a multivariate process that follows a normal distribution. In order to assess the proposed AEWMA-I multivariate dispersion chart, we analyze its RL profiles alongside the EWMA and AEWMA-II charts, considering various magnitudes of shift sizes. In our evaluation, we set the initial ARL (ARL_0_) to 370 and the smoothing constant (ψ) to 0.15. To calculate the run-length profiles of the AEWMA-I, AEWMA-II, and EWMA control charts, we conducted 50,000 iterations using the MC simulations method. This enables us to compare the performance of these control charts under different shift sizes.

### Comparison of proposed AEWMA-I and existing EWMA charts

**Figure 1:**
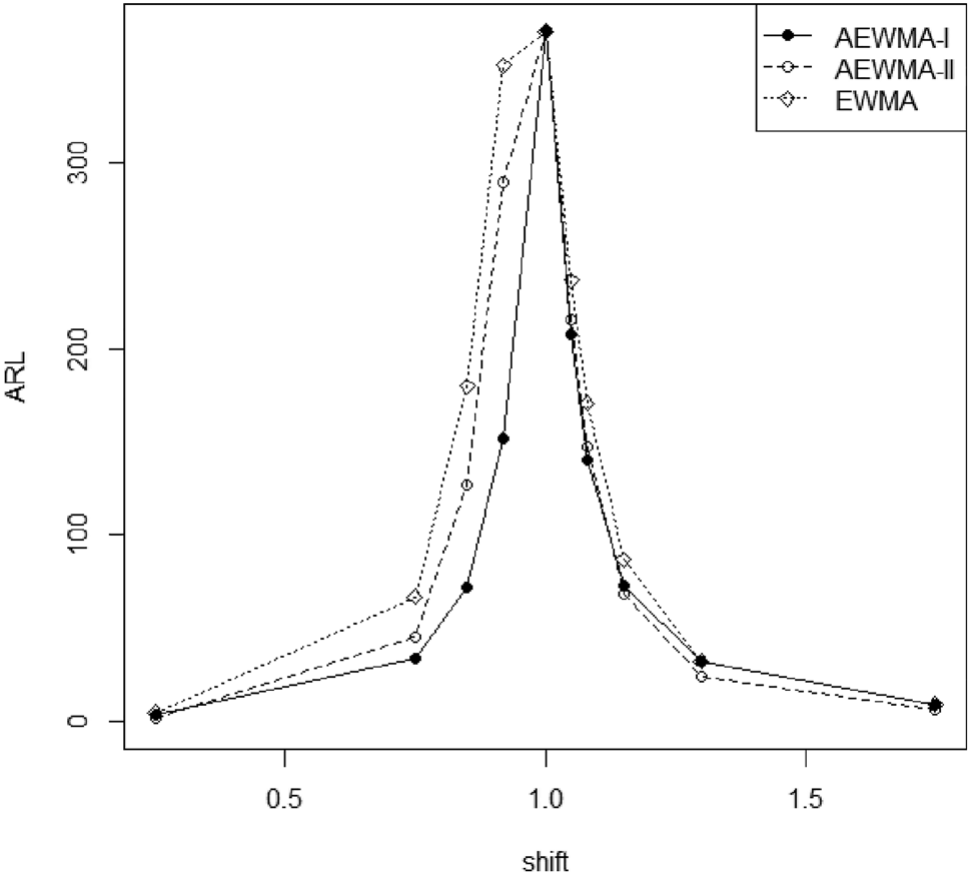
Comparison of the AEWMA-I, AEWMA-II, EWMA charts for *p* = 2.

**Figure 2:**
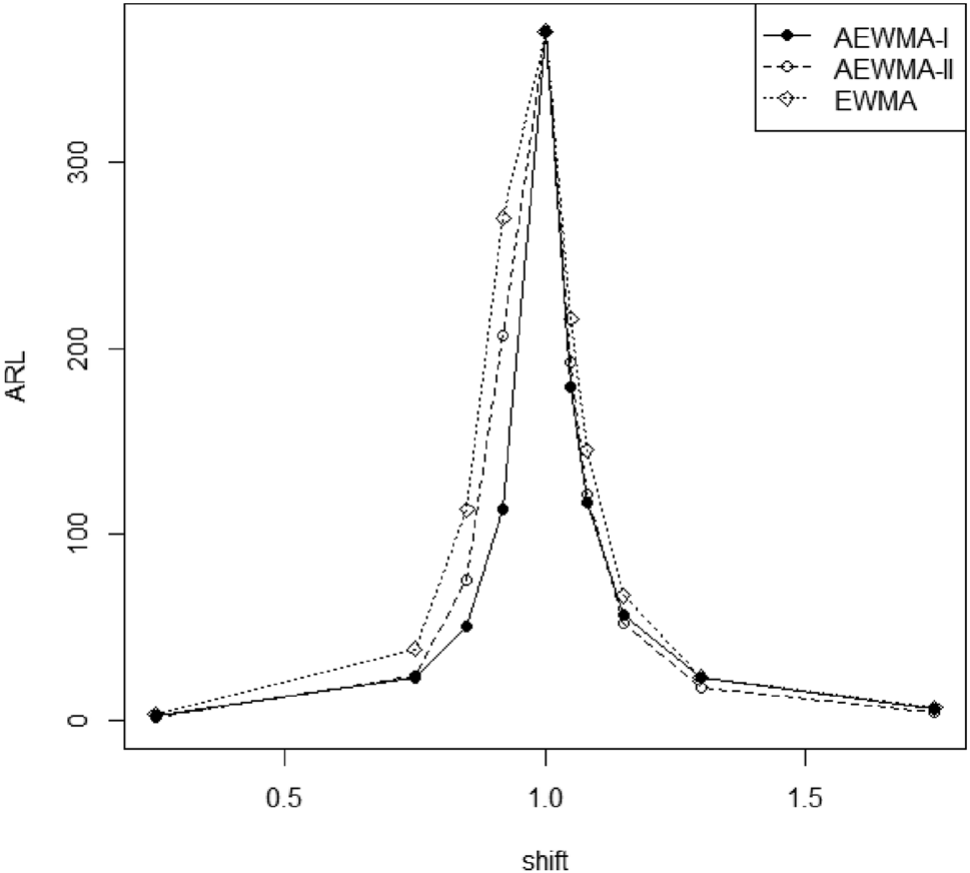
Comparison of the AEWMA-I, AEWMA-II, EWMA charts for *p* = 3.

**Figure 3:**
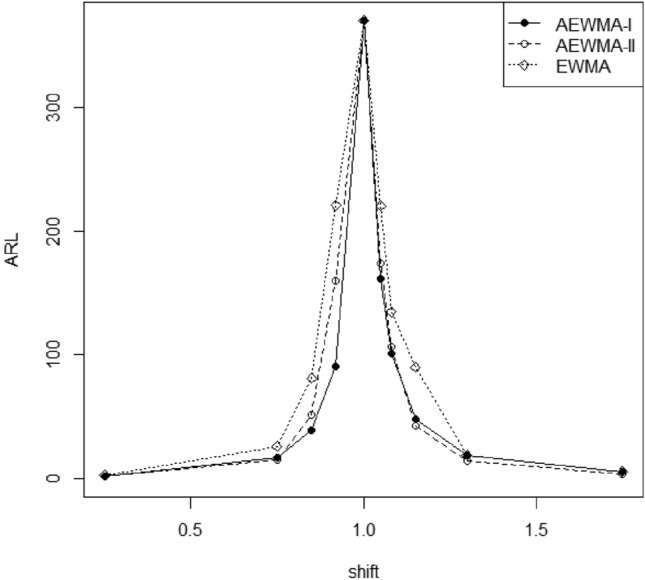
Comparison of the AEWMA-I, AEWMA-II, EWMA charts for *p* = 4.

**Figure 4:**
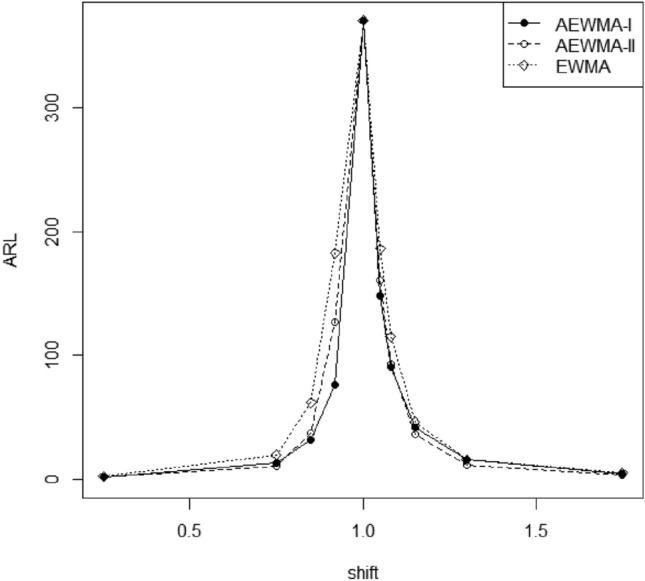
Comparison of the AEWMA-I, AEWMA-II, EWMA charts for *p* = 5.

The presentation of the AEWMA-I multivariate dispersion control chart with the EWMA chart is given at *p* = 2, 3, and 5 for $\delta$ in Tables 4, 5 and 6. The proposed one is efficient than the EWMA chart for detection of shifts in the covariance matrix. Furthermore, the out-of-control run-length profiles of the AEWMA-I control chart are are notably shorter compared to those of the EWMA control chart for all considered $\delta$ s, in other words, the AEWMA-I consistently enhances the run-length profiles compare to EWMA chart. The comparison between the proposed AEWMA-I multivariate dispersion control chart and the conventional EWMA chart was conducted for various values of *p*. The results are presented in Tables 4, 5 and 6 for different values of shifts. The results shows that the proposed control chart is efficient to detect the shifts in the covariance matrix as compared to the existing control chart. For example, at *p* = 2, the ARLs for $\delta$ = (0.80, 0.92, 1.05, 1.08) of the EWMA and AEWMA-I charts are (108.66, 351.54, 236.42, 170.77) and (48.14, 151.91, 207.36, 140.57), respectively. Similarly, at *p* = 2, the SDRLs for $\delta$ = (0.80, 0.92, 1.05, 1.08) of the EWMA and AEWMA-I charts are (101.03, 347.76, 231.21, 162.78) and (30.73, 121.29, 177.60, 111.18), correspondingly. One can infer from these observations that AEWMA-I chart is more reliable as compare to the EWMA control chart. The visually presented results in Figs. 1, 2, 3 and 4 also alines with the same findings.

**Table 4:** Comparative analysis of control charts based on run length profile.

δ		ARL	SDRL	P5th	P﻿10th	P25th	P50th	P75th	P90th	P95th
0.25	EWMA	4.98	1.20	3	4	4	5	6	7	7
	AEWMA-I	**3.98**	**1.15**	**2**	**2**	**3**	**4**	**5**	**5**	**6**
0.50	EWMA	12.37	6.41	5	6	8	11	15	21	25
	AEWMA-I	**9.33**	**4.69**	**4**	**4**	**6**	**8**	**12**	**16**	**18**
0.75	EWMA	67.14	59.83	10	14	25	49	90	144	186
	AEWMA-I	**34.43**	**21.57**	**6**	**9**	**18**	**31**	**47**	**64**	**75**
0.80	EWMA	108.66	101.03	12	18	37	78	149	240	310
	AEWMA-I	**48.14**	**30.73**	**8**	**12**	**25**	**44**	**66**	**90**	**105**
0.85	EWMA	180.11	172.48	16	26	57	127	248	405	525
	AEWMA-I	**71.97**	**48.03**	**10**	**18**	**37**	**64**	**97**	**136**	**163**
0.92	EWMA	351.54	347.76	23	42	104	245	483	802	1051
	AEWMA-I	**151.91**	**121.29**	**15**	**31**	**66**	**122**	**205**	**311**	**390**
0.95	EWMA	407.42	399.80	26	48	122	285	562	933	1206
	AEWMA-I	**237.34**	**213.39**	**18**	**38**	**88**	**177**	**322**	**515**	**658**
0.97	EWMA	420.31	414.69	27	49	124	292	581	962	1251
	AEWMA-I	**317.89**	**302.00**	**19**	**43**	**106**	**228**	**434**	**709**	**921**
1.00	EWMA	369.81	361.77	24	45	112	260	510	845	1094
	AEWMA-I	370.25	352.14	21	48	121	265	510	829	1073
1.03	EWMA	289.22	283.24	21	37	89	202	396	656	855
	AEWMA-I	**273.53**	**246.38**	**19**	**42**	**100**	**205**	**373**	**592**	**759**
1.05	EWMA	236.42	231.21	18	31	72	165	325	538	701
	AEWMA-I	**207.36**	**177.60**	**17**	**36**	**82**	**160**	**281**	**438**	**558**
1.08	EWMA	170.77	162.78	15	24	54	120	236	387	496
	AEWMA-I	**140.57**	**111.18**	**14**	**28**	**61**	**115**	**190**	**284**	**358**
1.10	EWMA	139.25	133.78	13	21	44	98	191	313	407
	AEWMA-I	**112.37**	**85.06**	**12**	**24**	**51**	**94**	**153**	**223**	**276**
1.15	EWMA	86.90	80.42	10	15	30	62	118	192	246
	AEWMA-I	**72.92**	**52.63**	**9**	**16**	**34**	**62**	**99**	**143**	**173**
1.30	EWMA	32.59	27.00	6	8	14	25	43	67	86
	AEWMA-I	**30.44**	**21.22**	**5**	**8**	**14**	**26**	**42**	**60**	**71**
1.75	EWMA	9.36	5.77	3	4	5	8	12	17	21
	AEWMA-I	**9.04**	**5.80**	**3**	**3**	**5**	**8**	**12**	**17**	**21**
3.50	EWMA	3.09	1.29	2	2	2	3	4	5	6
	AEWMA-I	**2.81**	**1.25**	**2**	**2**	**2**	**2**	**3**	**5**	**5**

Significant values are in bold.

**Table 5:** Comparative analysis based on run length profile for *p* = 3.

δ		ARL	SDRL	P5th	P10th	P25th	P50th	P75th	P90th	P95th
0.25	EWMA	3.70	**0.73**	**3**	**3**	**3**	**4**	**4**	**5**	**5**
	AEWMA-I	**2.82**	0.82	**2**	**2**	**2**	**3**	**3**	**4**	**4**
0.50	EWMA	8.03	3.31	4	5	6	7	10	12	14
	AEWMA-I	**6.16**	**2.69**	**2**	**3**	**4**	**6**	**7**	**10**	**11**
0.75	EWMA	38.51	31.40	8	10	16	29	51	79	100
	AEWMA-I	**23.19**	**14.94**	**5**	**7**	**11**	**20**	**32**	**44**	**51**
0.80	EWMA	63.14	56.44	9	13	24	46	85	136	174
	AEWMA-I	**33.40**	**21.89**	**6**	**8**	**16**	**30**	**46**	**63**	**75**
0.85	EWMA	113.56	106.91	12	18	38	81	155	253	327
	AEWMA-I	**50.82**	**34.21**	**7**	**11**	**25**	**45**	**70**	**97**	**115**
0.92	EWMA	269.94	262.35	19	34	82	190	373	611	797
	AEWMA-I	**113.31**	**87.33**	**10**	**21**	**50**	**94**	**155**	**228**	**282**
0.95	EWMA	363.10	356.22	23	43	108	254	501	831	1077
	AEWMA-I	**188.16**	**163.58**	**14**	**31**	**73**	**144**	**255**	**399**	**513**
0.97	EWMA	400.82	396.42	26	27	119	280	550	919	1189
	AEWMA-I	**276.88**	**260.64**	**16**	**38**	**94**	**200**	**379**	**618**	**795**
1.00	EWMA	370.72	362.45	24	44	111	260	514	845	1095
	AEWMA-I	370.37	358.69	19	46	117	262	511	838	1084
1.03	EWMA	276.17	269.44	20	35	85	194	379	629	817
	AEWMA-I	**252.80**	**222.24**	**18**	**40**	**95**	**193**	**346**	**541**	**689**
1.05	EWMA	215.87	209.42	17	29	67	152	295	491	631
	AEWMA-I	**179.51**	**146.03**	**16**	**33**	**75**	**144**	**243**	**371**	**467**
1.08	EWMA	145.53	138.30	14	21	47	103	199	325	420
	AEWMA-I	**116.82**	**86.65**	**13**	**25**	**54**	**98**	**159**	**232**	**284**
1.10	EWMA	115.24	107.31	12	19	38	83	157	257	329
	AEWMA-I	**92.22**	**66.20**	**11**	**21**	**44**	**79**	**126**	**180**	**219**
1.15	EWMA	67.53	60.38	9	13	25	49	91	147	188
	AEWMA-I	**57.17**	**39.31**	**8**	**14**	**28**	**50**	**78**	**110**	**132**
1.30	EWMA	23.52	17.99	5	7	11	18	31	47	59
	AEWMA-I	**22.94**	**15.24**	**5**	**7**	**11**	**20**	**31**	**44**	**52**
1.75	EWMA	7.14	3.86	3	3	4	6	9	12	15
	AEWMA-I	**6.87**	**3.91**	**3**	**3**	**4**	**6**	**9**	**12**	**15**
3.50	EWMA	2.59	0.85	**2**	**2**	**2**	**2**	**3**	**4**	**4**
	AEWMA-I	**2.39**	**0.78**	**2**	**2**	**2**	**2**	**3**	**3**	**4**

Significant values are in bold.

**Table 6:** Comparative analysis based on run length profile for *p* = 5.

δ		ARL	SDRL	P5th	P10th	P25th	P50th	P75th	P90th	P95th
0.25	EWMA	2.70	0.51	2	2	2	3	3	3	3
	AEWMA-I	**2.04**	**0.21**	**2**	**2**	**2**	**2**	**2**	**2**	**2**
0.50	EWMA	5.11	1.64	3	3	4	5	6	7	8
	AEWMA-I	**3.81**	**1.56**	**2**	**2**	**2**	**4**	**5**	**6**	**6**
0.75	EWMA	19.91	14.14	5	7	10	16	26	38	48
	AEWMA-I	**13.50**	**8.92**	**2**	**4**	**7**	**11**	**18**	**26**	**31**
0.80	EWMA	32.99	26.53	7	9	14	25	44	68	87
	AEWMA-I	**20.05**	**13.80**	**3**	**5**	**9**	**17**	**28**	**39**	**46**
0.85	EWMA	62.20	56.07	9	12	22	45	84	135	174
	AEWMA-I	**32.09**	**22.47**	**4**	**7**	**14**	**28**	**45**	**63**	**74**
0.92	EWMA	182.72	176.31	15	24	57	129	252	413	536
	AEWMA-I	**76.24**	**57.90**	**6**	**13**	**34**	**65**	**105**	**152**	**186**
0.95	EWMA	293.04	289.29	19	35	87	204	407	671	870
	AEWMA-I	**134.43**	**114.47**	**8**	**19**	**52**	**106**	**184**	**285**	**360**
0.97	EWMA	362.89	359.34	23	42	106	250	504	830	1082
	AEWMA-I	**219.94**	**206.33**	**10**	**27**	**75**	**162**	**302**	**487**	**625**
1.00	EWMA	369.30	363.54	24	44	110	258	511	838	1090
	AEWMA-I	370.21	363.32	14	40	112	261	514	843	1084
1.03	EWMA	258.03	250.42	20	33	79	180	356	588	756
	AEWMA-I	**222.06**	**187.51**	**15**	**38**	**90**	**174**	**303**	**466**	**589**
1.05	EWMA	186.20	179.75	17	27	59	131	254	418	545
	AEWMA-I	**147.86**	**112.34**	**14**	**30**	**67**	**123**	**201**	**296**	**364**
1.08	EWMA	115.32	108.31	13	19	39	82	157	254	331
	AEWMA-I	**90.42**	**62.92**	**12**	**21**	**45**	**78**	**122**	**173**	**209**
1.10	EWMA	85.47	77.86	11	16	30	62	115	188	243
	AEWMA-I	**69.65**	**46.95**	**10**	**17**	**35**	**61**	**94**	**132**	**159**
15	EWMA	46.73	39.49	8	11	19	35	62	97	126
	AEWMA-I	**42.16**	**27.39**	**8**	**11**	**21**	**37**	**57**	**79**	**94**
1.30	EWMA	16.04	10.69	5	6	9	13	21	30	37
	AEWMA-I	**16.06**	**10.03**	**4**	**6**	**9**	**14**	**21**	**30**	**36**
1.75	EWMA	5.25	2.34	2	3	4	5	6	8	10
	AEWMA-I	**5.03**	**2.43**	**2**	**2**	**3**	**5**	**6**	**8**	**10**
3.50	EWMA	2.21	0.47	2	2	2	2	2	3	3
	AEWMA-I	**2.10**	**0.36**	**2**	**2**	**2**	**2**	**2**	**2**	**3**

Significant values are in bold.

### Comparison of proposed AEWMA-I and existing AEWMA-II charts

In Tables 7, 8 and 9, we presented th comparison of AEWMA-I and AEWMA-II charts. The AEWMA-I performs better than AEWMA-II at the various shift sizes $\delta \in$[0.75, 1.10]. It's important to highlight that the AEWMA-II chart exhibits a notably poor performance in terms of SDRLs.The SDRLs of the AEWMA-II chart are greater than those of ARLs. That’s why when $\delta \in \left(\left[\mathrm{0.25,0.50}\right]\wedge \left[\mathrm{1.15,1.75}\right]\right)$ AEWMA-II chart seems a bit better than the AEWMA-I chart and otherwise, the effectiveness of both charts is the same. For example, at *p* = 2, the ARLs for $\delta$ = (1.05, 1.15, 3.50) of the AEWMA-II and AEWMA-I charts are (215.76, 68.86, 2.36) and (207.36, 72.92, 2.81), respectively. Similarly, at *p* = 2, the SDRLs for $\delta$ = (1.05, 1.15, 3.50) of the existing AEWMA-II and AEWMA-I charts are (236.20, 71.47, 0.87) and (177.60, 52.63, 1.25), respectively. Overall, the results from Tables 7, 8 and 9 suggest that the AEWMA-I control chart is generally superior to the AEWMA-II control chart in terms of percentiles, indicating better early detection of process shifts. However, the AEWMA-II control chart may have a slight advantage in terms of ARLs for moderate and large shifts, though its results might be less stable than those of the AEWMA-I control chart. Also, it can be seen that P10 = (5, 5) and P95 = (1326, 886) in AEWMA-II control chart whereas P10 = (43, 42) and P95 = (921, 759) in AEWMA-I control chart at δ = (0.97, 1.03). These observations aligns with our findings in the run-length profile results, particularly at p = 3 and 5. Additionally, these findings are visually reinforced in Figs. 1, 2, 3 and 4.

**Table 7:** Comparative analysis based on run length profile.

δ		ARL	SDRL	P5th	P10th	P25th	P50th	P75th	P90th	P95th
0.25	AEWMA-II	**2.30**	**0.77**	**2**	**2**	**2**	**2**	**2**	**3**	**4**
	AEWMA-I	3.98	1.15	2	2	3	4	5	5	6
0.50	AEWMA-II	**6.99**	6.03	2	2	2	5	9	15	19
	AEWMA-I	9.33	**4.69**	**4**	**4**	**6**	**8**	**12**	**16**	**18**
0.75	AEWMA-II	45.53	49.31	2	2	9	30	65	110	145
	AEWMA-I	**34.43**	**21.57**	**6**	**9**	**18**	**31**	**47**	**64**	**75**
0.80	AEWMA-II	74.84	82.11	2	2	14	49	107	182	239
	AEWMA-I	**48.14**	**30.73**	**8**	**12**	**25**	**44**	**66**	**90**	**105**
0.85	AEWMA-II	126.63	140.00	2	3	23	83	181	309	408
	AEWMA-I	**71.97**	**48.03**	**10**	**18**	**37**	**64**	**97**	**136**	**163**
0.92	AEWMA-II	289.50	326.00	2	4	52	187	413	716	944
	AEWMA-I	**151.91**	**121.29**	**15**	**31**	**66**	**122**	**205**	**311**	**390**
0.95	AEWMA-II	377.24	423.62	2	5	68	241	540	933	1226
	AEWMA-I	**237.34**	**213.39**	**18**	**38**	**88**	**177**	**322**	**515**	**658**
0.97	AEWMA-II	408.78	458.50	2	5	74	263	587	1004	1326
	AEWMA-I	**317.89**	**302.00**	**19**	**43**	**106**	**228**	**434**	**709**	**921**
1.00	AEWMA-II	370.21	414.71	2	5	68	238	529	917	1210
	AEWMA-I	**370.25**	**352.14**	**21**	**48**	**121**	**265**	**510**	**829**	**1073**
1.03	AEWMA-II	274.75	306.19	2	5	53	177	390	676	886
	AEWMA-I	**273.53**	**246.38**	**19**	**42**	**100**	**205**	**373**	**592**	**759**
1.05	AEWMA-II	215.76	236.20	2	5	45	142	307	527	688
	AEWMA-I	**207.36**	**177.60**	**17**	**36**	**82**	**160**	**281**	**438**	**558**
1.08	AEWMA-II	147.00	159.79	2	5	31	97	209	355	467
	AEWMA-I	**140.57**	**111.18**	**14**	**28**	**61**	**115**	**190**	**284**	**358**
1.10	AEWMA-II	115.53	124.38	2	4	25	77	163	281	367
	AEWMA-I	**112.37**	**85.06**	**12**	**24**	**51**	**94**	**153**	**223**	**276**
1.15	AEWMA-II	**68.86**	71.47	2	3	16	47	98	163	211
	AEWMA-I	72.92	**52.63**	**9**	**16**	**34**	**62**	**99**	**143**	**173**
1.30	AEWMA-II	24.25	23.58	2	2	7	17	34	55	71
	AEWMA-I	30.44	**21.22**	**5**	**8**	**14**	**26**	**42**	**60**	**71**
1.75	AEWMA-II	**6.47**	**5.26**	**2**	**2**	**2**	**5**	**9**	**13**	**17**
	AEWMA-I	9.04	5.90	2	3	5	8	12	17	21
3.50	AEWMA-II	**2.36**	**0.87**	**2**	**2**	**2**	**2**	**2**	**3**	**4**
	AEWMA-I	2.81	1.25	2	2	2	2	3	5	5

Significant values are in bold.

**Table 8:** Comparative analysis based on run length profile for p = 3.

δ		ARL	SDRL	P5th	P10th	P25th	P50th	P75th	P90th	P95th
0.25	AEWMA-II	**2.01**	**0.13**	2	2	2	**2**	**2**	**2**	**2**
	AEWMA-I	2.82	0.82	2	2	2	3	3	4	4
0.50	AEWMA-II	**3.84**	2.82	2	2	2	2	5	8	10
	AEWMA-I	6.16	**2.69**	**2**	**3**	**4**	**6**	**7**	**10**	**11**
0.75	AEWMA-II	24.18	26.83	2	2	4	15	35	60	78
	AEWMA-I	**23.19**	**14.94**	**5**	**7**	**11**	**20**	**32**	**44**	**51**
0.80	AEWMA-II	41.77	47.15	2	2	7	26	60	103	136
	AEWMA-I	**33.40**	**21.89**	**6**	**8**	**16**	**30**	**46**	**63**	**75**
0.85	AEWMA-II	75.84	87.21	2	2	11	48	108	188	249
	AEWMA-I	**50.82**	**34.21**	**7**	**11**	**25**	**45**	**70**	**97**	**115**
0.92	AEWMA-II	206.90	241.65	2	2	30	128	295	519	688
	AEWMA-I	**113.31**	**87.33**	**10**	**21**	**50**	**94**	**155**	**228**	**282**
0.95	AEWMA-II	315.57	364.54	2	3	48	198	453	791	1041
	AEWMA-I	**188.16**	**163.58**	**14**	**31**	**73**	**144**	**255**	**399**	**513**
0.97	AEWMA-II	377.05	432.48	2	3	60	237	545	941	1238
	AEWMA-I	**276.88**	**260.64**	**16**	**38**	**94**	**200**	**379**	**618**	**795**
1.00	AEWMA-II	369.24	420.27	2	4	63	235	527	918	1208
	AEWMA-I	370.37	**358.69**	**19**	**46**	**117**	**262**	**511**	**838**	**1084**
1.03	AEWMA-II	261.05	291.31	2	4	48	170	374	644	838
	AEWMA-I	**252.80**	**222.24**	**18**	**40**	**95**	**193**	**346**	**541**	**689**
1.05	AEWMA-II	192.61	212.98	2	4	38	125	273	470	619
	AEWMA-I	**179.51**	**146.03**	**16**	**33**	**75**	**144**	**243**	**371**	**467**
1.08	AEWMA-II	121.26	130.09	2	4	27	82	171	292	381
	AEWMA-I	**116.82**	**86.65**	**13**	**25**	**54**	**98**	**159**	**232**	**284**
1.10	AEWMA-II	93.54	98.67	2	4	22	64	132	221	289
	AEWMA-I	**92.22**	**66.20**	**11**	**21**	**44**	**79**	**126**	**180**	**219**
1.15	AEWMA-II	**52.80**	52.95	2	3	14	38	74	122	159
	AEWMA-I	57.17	**39.31**	**8**	**14**	**28**	**50**	**78**	**110**	**132**
1.30	AEWMA-II	**17.78**	16.17	2	2	6	13	25	39	50
	AEWMA-I	22.94	**15.24**	**5**	**7**	**11**	**20**	**31**	**44**	**52**
1.75	AEWMA-II	**4.91**	**3.58**	**2**	**2**	**2**	**4**	**6**	**10**	**12**
	AEWMA-I	6.87	3.96	2	3	4	6	9	12	15
3.50	AEWMA-II	**2.13**	**0.47**	**2**	**2**	**2**	**2**	**2**	**2**	**3**
	AEWMA-I	2.39	0.78	2	2	2	2	3	3	4

Significant values are in bold.

**Table 9:** Comparative analysis based on run length profile for *p* = 5.

δ		ARL	SDRL	P5th	P10th	P25th	P50th	P75th	P90th	P95th
0.25	AEWMA-II	**2.00**	**0.00**	**2**	**2**	**2**	**2**	**2**	**2**	**2**
	AEWMA-I	2.04	0.21	**2**	**2**	**2**	**2**	**2**	**2**	**2**
0.50	AEWMA-II	**2.32**	**0.90**	**2**	**2**	**2**	**2**	**2**	**3**	**4**
	AEWMA-I	3.81	1.56	2	2	2	4	5	6	6
0.75	AEWMA-II	**10.73**	12.25	2	2	2	6	15	27	36
	AEWMA-I	13.50	**8.92**	**2**	**4**	**7**	**11**	**18**	**26**	**31**
0.80	AEWMA-II	**19.11**	22.68	2	2	2	10	27	49	65
	AEWMA-I	20.05	**13.80**	**3**	**5**	**9**	**17**	**28**	**39**	**46**
0.85	AEWMA-II	37.41	45.52	2	2	4	21	54	97	130
	AEWMA-I	**32.09**	**22.47**	**4**	**7**	**14**	**28**	**45**	**63**	**74**
0.92	AEWMA-II	127.07	154.70	2	2	11	74	183	329	442
	AEWMA-I	**76.24**	**57.90**	**6**	**13**	**34**	**65**	**105**	**152**	**186**
0.95	AEWMA-II	232.96	280.30	2	2	23	139	338	597	800
	AEWMA-I	**134.43**	**114.47**	**8**	**19**	**52**	**106**	**184**	**285**	**360**
0.97	AEWMA-II	328.84	390.18	2	2	36	200	474	840	1121
	AEWMA-I	**219.94**	**206.33**	**10**	**27**	**75**	**162**	**302**	**487**	**625**
1.00	AEWMA-II	369.19	430.74	2	2	50	227	532	939	1237
	AEWMA-I	370.21	**363.32**	**14**	**40**	**112**	**261**	**514**	**843**	**1084**
1.03	AEWMA-II	238.93	270.50	2	3	41	151	344	594	780
	AEWMA-I	**222.06**	**187.51**	**15**	**38**	**90**	**174**	**303**	**466**	**589**
1.05	AEWMA-II	160.94	176.94	2	3	33	106	229	388	510
	AEWMA-I	**147.86**	**112.34**	**14**	**30**	**67**	**123**	**201**	**296**	**364**
1.08	AEWMA-II	93.57	98.81	2	3	22	64	132	222	290
	AEWMA-I	**90.42**	**62.92**	**12**	**21**	**45**	**78**	**122**	**173**	**209**
1.10	AEWMA-II	**68.66**	69.90	2	3	17	49	97	159	207
	AEWMA-I	69.65	**46.95**	**10**	**17**	**35**	**61**	**94**	**132**	**159**
1.15	AEWMA-II	**36.65**	35.06	2	3	11	27	51	82	106
	AEWMA-I	42.16	**27.39**	**8**	**11**	**21**	**37**	**57**	**79**	**94**
1.30	AEWMA-II	**12.25**	10.19	2	2	5	10	17	26	32
	AEWMA-I	16.14	**10.03**	**4**	**6**	**9**	**14**	**21**	**30**	**36**
1.75	AEWMA-II	**3.56**	**2.15**	**2**	**2**	**2**	**3**	**5**	**7**	**8**
	AEWMA-I	5.03	2.48	2	2	3	5	6	8	10
3.50	AEWMA-II	**2.02**	**0.16**	**2**	**2**	**2**	**2**	**2**	**2**	**2**
	AEWMA-I	2.10	0.36	2	2	2	2	2	2	3

Significant values are in bold.

## Illustrative example

The real dataset used in the study is taken from Santos-Fernández^28^. The dataset pertains to a bimetal thermostat, a device commonly used for various practical applications. Bimetal thermostats utilize a bimetallic strip composed of two different metallic strips. This bimetallic strip converts temperature changes into mechanical displacement due to the varying thermal expansion properties of the two metals. In this study, the bimetallic strip, made by combining steel and brass metals, is subjected to quality testing in a laboratory. The bimetallic strip, got by joining steel and brass metals, is investigated in a quality testing lab by testing five quality attributes, including, the redirection (V1), curvature (V2), resistivity (V3), hardness in the low expansion side (V4) and hardness in high expansion side (V5). The quality control division takes 28 samples from the assembling process for both Phase-I and Phase-II datasets. The Phase-I dataset is used to estimate the parameter of the process, as the parameter of the process is unknown, and the twenty-eight samples of Phase-II are considered to observe the covariance matrix of the process.

**Figure 5:**
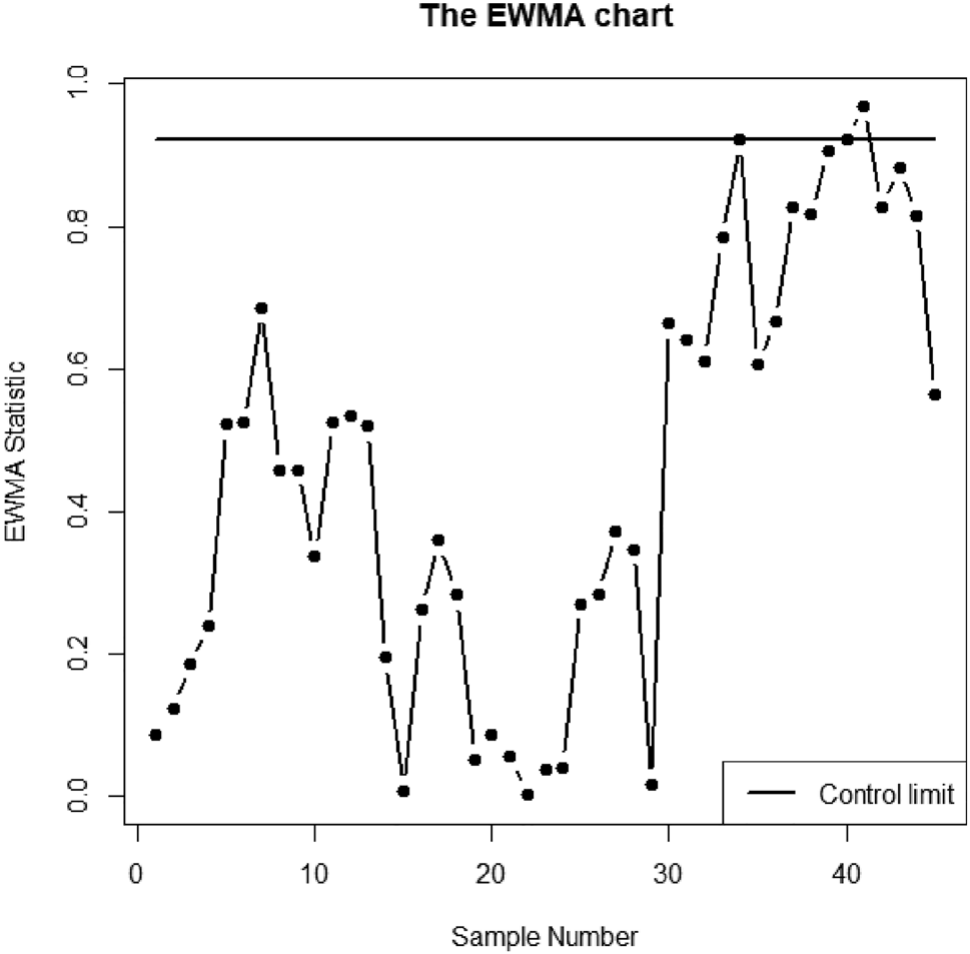
The EWMA chart for bimetal thermostat data.

**Figure 6:**
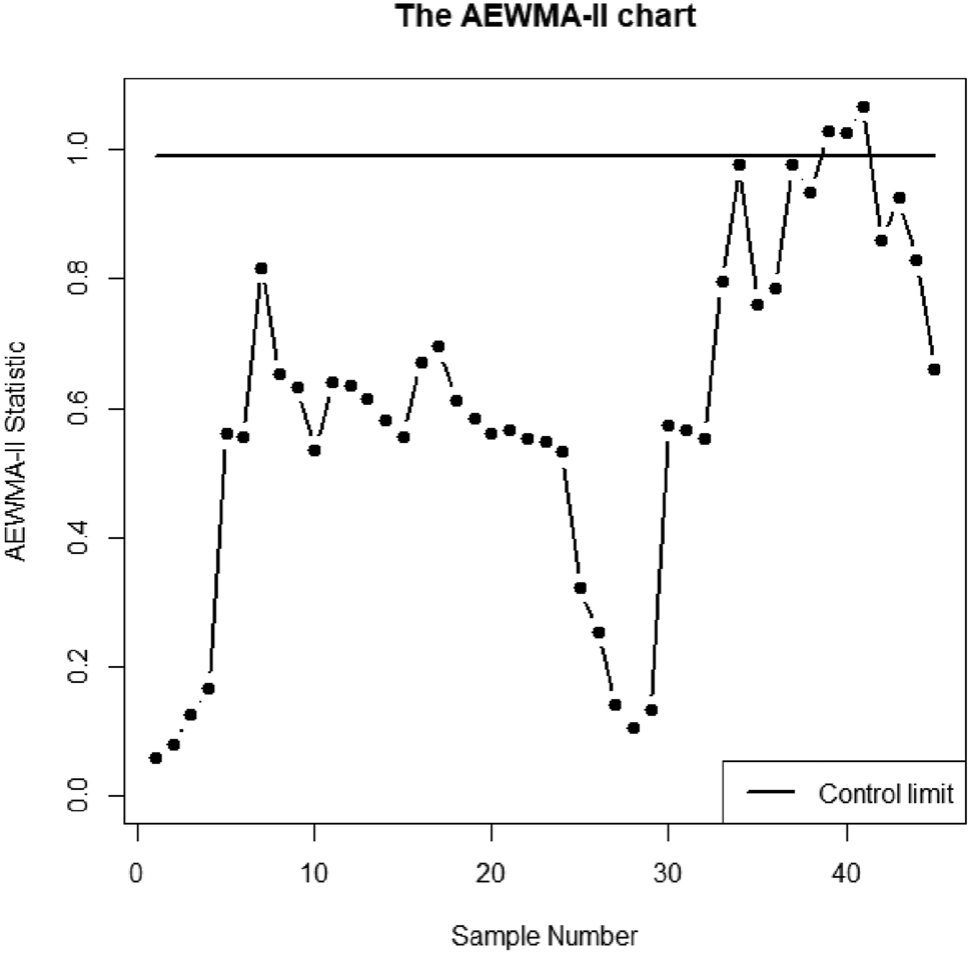
The AEWMA-II chart for bimetal thermostat data.

**Figure 7:**
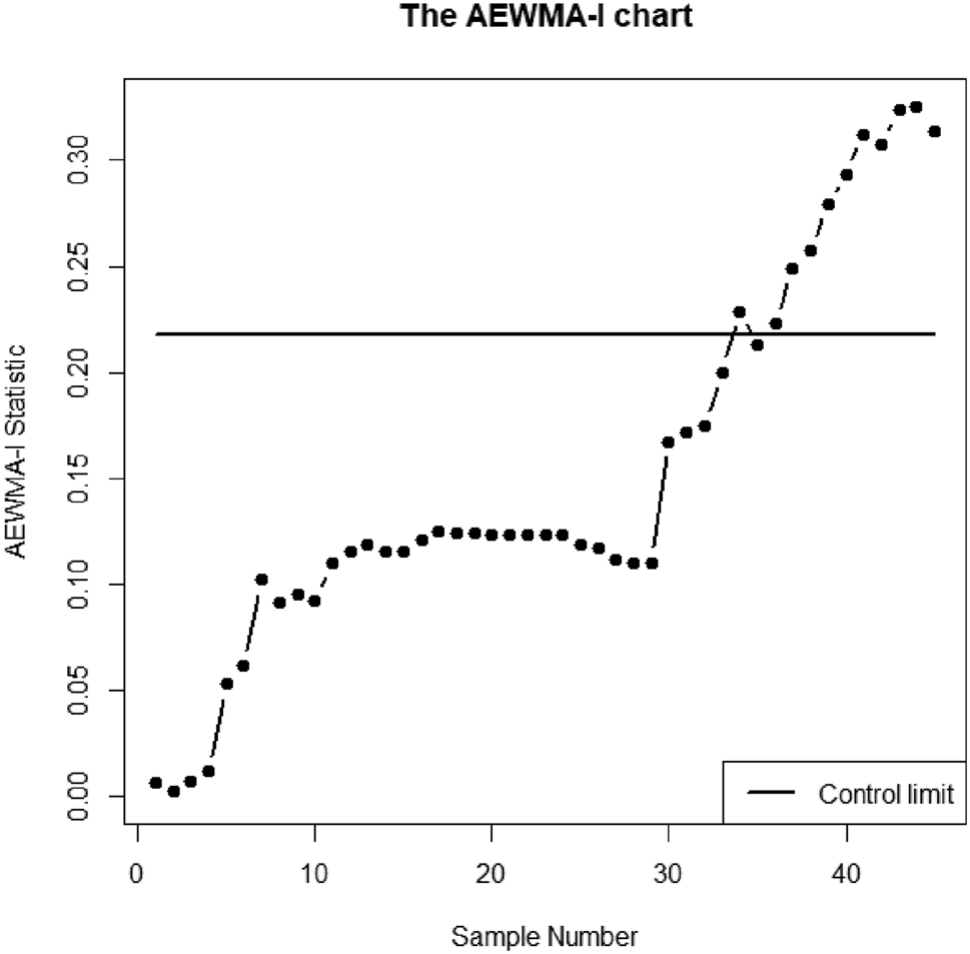
The AEWMA-I chart for bimetal thermostat data.

Here, the understudy quality characteristics variables are V1, V4, and V5 which is *p* = 3. The proposed AEWMA-I, AEWMA-II, and EWMA control charts are applied to this dataset using in-control ARL as 370. The parametric choice for proposed AEWMA-I, AEWMA-II, and EWMA control charts are (*L* = 0.2181, $\psi$=0.15), (*L* = 0.9908, $\psi$=0.15) and (*L* = 0.9215, $\psi$=0.15) respectively. Figs. 5, 6 and 7 display the proposed AEWMA-I, AEWMA-II, and EWMA control charts. The process parameter estimation is as follows

$${\widehat{{\varvec{\Sigma}}}}_{0}=\left[\begin{array}{ccc}0.030018386 & 0.011894709 & 0.008731614\\ 0.011894709 & 0.039277249 & 0.009142328\\ 0.008731614 & 0.009142328 & 0.021699868\end{array}\right]$$

Figures 5, 6 and 7 make it clear that all three control charts have remained stable for the first 28 samples, indicating that the process is currently under control. Nevertheless, all three charts in the subsequent 28 samples demonstrate an ascending change in the process covariance matrix. The EWMA, AEWMA-II, and AEWMA-I control charts create out-of-control signals at the 40th, 39th, and 34th observations, respectively. An intriguing observation is that the AEWMA-I control chart provides an out-of-control signal earlier compared to the EWMA and AEWMA-II control charts. This illustrates the superiority of the proposed control chart over the multivariate control charts under consideration.

The proposed AEWMA-I control chart offers the advantage of early detection of shifts in the covariance matrix of the process compared to existing control charts. This early detection enables the identification of process variations at an earlier stage, resulting in fewer defective items being produced. Consequently, this leads to cost savings by reducing the expenses associated with discarding faulty products and the cost of reworking them. Moreover, when monitoring correlated multivariate data, using a single multivariate control chart is more appropriate and cost-effective compared to employing multiple univariate charts for each quality characteristic. This becomes particularly relevant when there are numerous related quality characteristics to be monitored. Overall, the proposed AEWMA-I control chart demonstrates higher efficiency than its counterparts in promptly generating out-of-control signals, allowing for timely intervention and quality improvement in the production process.

## Conclusions and further recommendations

Recently, adaptive control charts have gained significant attention due to their increased sensitivity compared to non-adaptive control charts. They are particularly useful in providing better protection when the process shift is expected to occur within a certain range. We proposed the AEWMA-I multivariate dispersion control chart as a method to monitor irregular variations in the covariance matrix of a process following a normal distribution. The MC simulation method is used to compute the average run length (ARL) for performance evaluation. Through comprehensive analysis of ARL properties, we find that the AEWMA-I control chart consistently outperforms other memory-based control charts in detecting variations in the covariance matrix of the process. Furthermore, the AEWMA-I control chart exhibits a smaller standard deviation of run length (SDRL) values, making it more reliable for real-life applications. To illustrate its application, we provide a numerical example using real-life data. Thus, we recommend using the AEWMA-I control chart for monitoring irregular variations in the covariance matrix of multivariate processes following a normal distribution.

In future research, it would be valuable to develop new AEWMA charts that monitor shifts in the process mean vector or jointly monitor both the mean vector and covariance matrix. Additionally, extending the current research to design AEWMA control charts for non-normally distributed processes would be an interesting avenue to explore. Another important area of investigation could involve understanding the causes behind signals generated by control charts for multivariate data, particularly when monitoring a process covariance matrix. The theoretical contribution behind the proposed dispersion control chart is the target to provide a sensitive control chart with not only gives quick detection of dispersion shift but also improves the SDRL characteristic in comparison with the existing AEWMA-II dispersion control chart. The respective suggested design with controlled SDRL and improved ARL would open new practical implications to utilize the design and may give manufacturing process defect free environment. The SPC literature is not as much enriched with multivariate dispersion adaptive designs to practically suggest designs to real life industries. So, the proposed AEWMA-I multivariate dispersion control chart would be a remarkable effort in this regard as the manufacturer is more comfortable utilizing multiple variables monitoring through a single plotting statistic rather than a univariate.

## Data Availability

The datasets used for this study can be requested from the corresponding author on reasonable request. No experiments involving human subjects or the utilization of human tissue samples were conducted in this study.

## Abbreviations

SPC: Statistical process control
MC: Monte Carlo
CUSUM: Cumulative SUM
DCUSUM: Dual cumulative SUM
EWMA: Exponential weighted moving average
MEWMA: Multivariate exponential weighted moving average
AEWMA: Adaptive EWMA
RL: Run length
ARL: Average run length
SDRL: Standard deviation of RL
ARL0: In control/initial ARL some pre decided fixed level
